# A novel classification of HCC basing on fatty-acid-associated lncRNA

**DOI:** 10.1038/s41598-022-23681-0

**Published:** 2022-11-07

**Authors:** Yating Xu, Xiao Yu, Qiyao Zhang, Yuting He, Wenzhi Guo

**Affiliations:** 1 Department of Hepatobiliary and Pancreatic Surgery, The First Affiliated Hospital of Zhengzhou University, Zhengzhou, 450052 People’s Republic of China; 2 Key Laboratory of Hepatobiliary and Pancreatic Surgery and Digestive Organ Transplantation of Henan Province, The First Affiliated Hospital of Zhengzhou University, Zhengzhou, China; 3 Open and Key Laboratory of Hepatobiliary & Pancreatic Surgery and Digestive Organ Transplantation at Henan Universities, Zhengzhou, China; 4 Henan Key Laboratory of Digestive Organ Transplantation, Zhengzhou, China

**Keywords:** Cancer, Biomarkers

## Abstract

Aberrant long noncoding RNA (lncRNA) expression and fatty acid signaling dysfunction both contribute to hepatocellular carcinoma (HCC) occurrence and development. However, the relationship and interaction mechanism between lncRNAs and fatty acid signaling in HCC remain unclear. Data regarding RNA expression and clinical outcomes for patients with HCC were obtained from The Cancer Genome Atlas (TCGA), HCCDB, and the Gene Expression Omnibus (GEO) databases. Hallmark pathways were identified using the single-sample gene set enrichment analysis (ssGSEA) method. ConsensusClusterPlus was used to establish a consistency matrix for classifying samples into three subtypes. A risk signature was established, and predictive values for key lncRNAs related to prognosis were evaluated using Kaplan–Meier analysis and receiver operating characteristic curves. The ESTIMATE algorithm, MCP-Counter, and ssGSEA were used to evaluate the characteristics of the tumor immune microenvironment. The CTRP2.0 and PRISM were used to analyze drug sensitivity in HCC subtypes. We discovered seven fatty-acid-associated lncRNAs with predictive prognostic capabilities, including *TRAF3IP2*-*AS1*, *SNHG10*, *AL157392*.2, *LINC02641*, *AL357079*.*1*, *AC046134.2*, and *A1BG-AS*. Three subtypes were obtained, which presented with differences in prognosis, clinical information, mutation features, pathway traits, immune characteristics, and drug sensitivity. The seven key lncRNAs identified in this study might serve as promising biomarkers for predicting prognosis in patients with HCC, and the three HCC subtypes classified according to lncRNA expression profiles could improve HCC classification.

## Introduction

Among malignant tumor types, liver cancer is one of the most predominant causes of death, with a global incidence of fatality that ranges from 600,000 to 800,000 per year^1^. Hepatocellular carcinoma (HCC) is the most commonly encountered type of liver cancer, accounting for approximately 80–90% of all liver cancer cases^2^. Various therapeutic strategies have emerged for HCC, including traditional surgery, radiotherapy, chemotherapy, vascular interventional treatments, and immunotherapy^3^. However, the 5-year survival rate among HCC patients remains unsatisfactory due to a tendency for patients to be diagnosed after reaching an advanced stage^4^. Therefore, further exploration of the underlying pathogenesis of HCC and the identification of early diagnostic biomarkers are necessary steps to improve the currently dismal prognosis.

Long noncoding RNAs (lncRNAs), which are described as RNAs longer than 200 nucleotides in length and do not encode protein product, have recently attracted a great deal of attention^5^. Numerous studies have revealed that lncRNAs participate in a variety of biological processes by mediating gene expression^6–8^. Abnormal lncRNA expression impacts the initiation and development of various diseases^9–11^. The lncRNA *PKMYT1AR* has been shown to sponge miR-485-5p, promoting PKMYT1 expression and supporting the maintenance of cancer stem cells during non-small cell lung cancer^12^. In HCC tissues, the lncRNA *TSLNC8* expressed in lower levels than in normal tissues, and the upregulation of *TSLNC8* was shown to prevent proliferation and metastasis in HCC^13^, suggesting its potential for a prognostic predictor.

Increasing evidence has revealed that metabolic dysregulation serves as a hallmark of tumorigenesis and progression in malignant tumors^14,15^. In particular, lipid metabolic reprogramming is found to significantly alter in cancer cells. Fatty acid (FA) accumulation plays crucial roles in membrane synthesis, energy storage, and the generation of signaling molecules. Aberrant FA oxidation may be significantly involved in the pathogenic mechanisms of multiple cancers, including gastric cancer (GC)^13^ and breast cancer (BC)^16^. The lncRNA *AGAP2-AS1* is thought to modulate FA oxidation to promote trastuzumab resistance in BC^17,18^. In GC, mesenchymal stem cells decreased the drug sensitivity of cancer cells due to the effects on FA oxidation mediated by increased lncRNA *MACC1-AS1* expression^19,20^. However, the associations between lncRNAs and FA pathways in HCC remain uncharted.

In our study, gene expression profiles and clinical data for patients with HCC were obtained from The Cancer Genome Atlas (TCGA), Gene Expression Omnibus (GEO) and HCCDB databases. We used bioinformatics tools to identify corresponding FA pathways and detected seven FA-associated lncRNAs related to HCC prognosis. Samples from two HCC cohorts were classified into three molecular subtypes based on the expression patterns of these seven lncRNAs. We further explored the biological characteristics and clinical significance of these newly defined molecular subtypes. These findings contribute to the current understanding of the relationships between lncRNAs and FA signaling in HCC and provide guidance for significantly prolonging HCC prognosis.

## Results

### Identification of FA-associated lncRNAs

**Figure 1:**
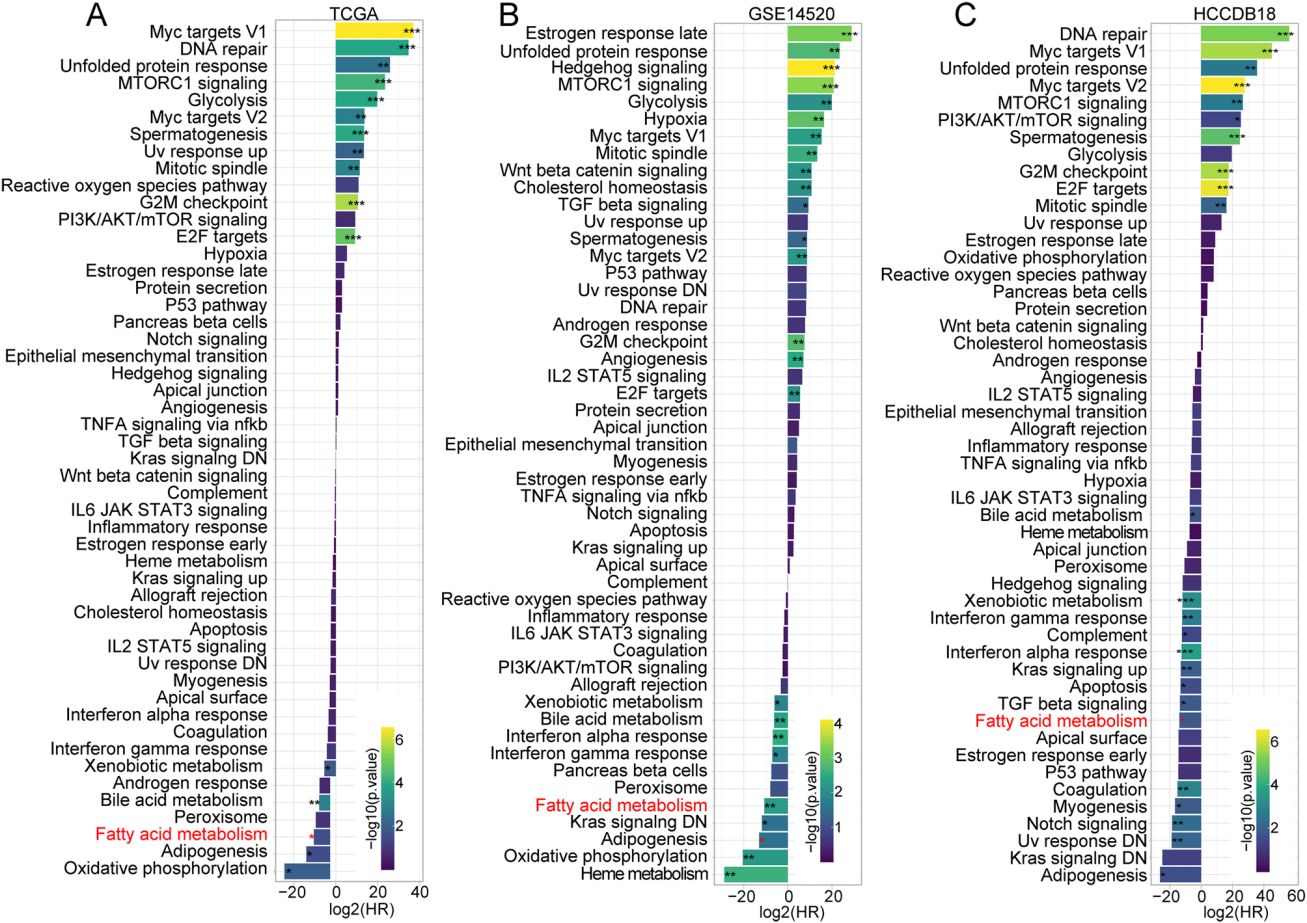
Hallmark pathways associated with prognosis (**A**) Univariate analysis of hallmark pathways in the TCGA database. (**B**) Univariate analysis of hallmark pathways in the GSE14520 database. (**C**) Univariate analysis of hallmark pathways in the HCCDB18 database.

**Figure 2:**
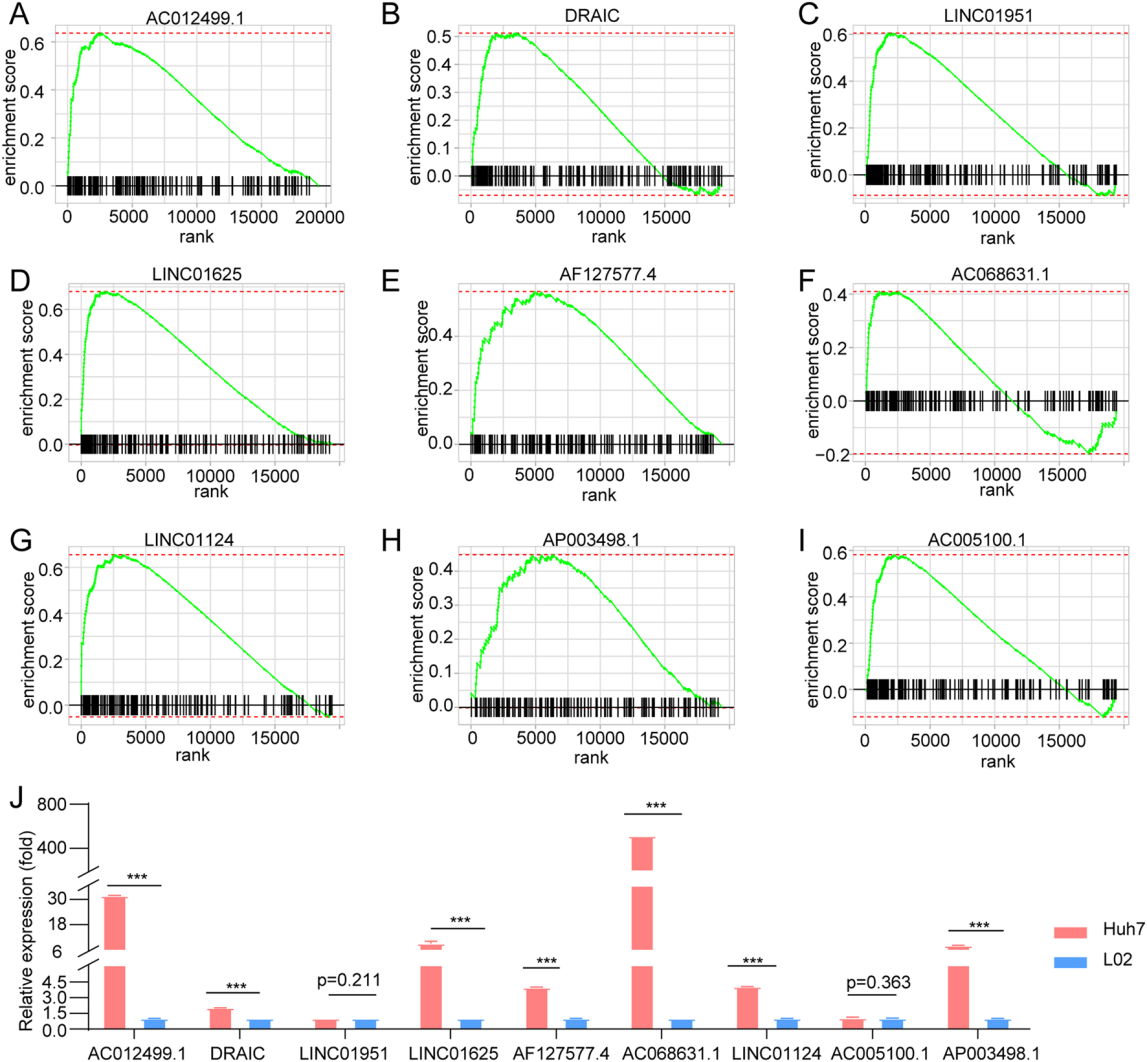
Validation of fatty acid-associated long noncoding RNAs (lncRNA)s. (**A**–**I**) Graphical display of the gene set enrichment analysis (GSEA) for top nine fatty acid-associated lncRNAs in fatty acid pathways (TCGA-LIHC). (**J**) Relative expression levels of nine lncRNAs in Huh7 and L02 cell lines.

We adopted the ssGSEA method to calculate hallmark pathway scores for samples obtained from the TCGA, HCCDB18, and GSE14520 datasets, followed by univariate Cox analyses to identify significant hallmark pathways associated with prognosis. The log2 (HR) > 0 represents the risk factor, and the log2 (HR) < 0 represents the protective factor. FA-metabolism signaling in the TCGA, GSE14520 datasets, and HCCDB18 showed a significant relationship with prognosis and served as a protective factor in HCC (Fig. 1). The number of lncRNA in HCCDB18 as well as in the GSE14520 dataset was less than 100, so we focused on TCGA and GSE76427 to identified corresponding lncRNA. Using the methods described in the “Materials and methods” section, 155 lncRNAs in the TCGA dataset and 663 lncRNAs in the GSE76427 datasets were identified as FA-associated lncRNAs, indicating an unsatisfactory consistency in the detection of lncRNAs associated with FA activity between datasets obtained from different platforms. The intersection of the TCGA and GSE764277 datasets contained 75 lncRNAs (Supplementary Fig. 1A). There are numerous lncRNAs positively enriched at the top of their respective ordered gene lists (Fig. 2A–I), and we selected top nine lncRNAs to exhibit and detected nine lnRNAs expression in L02 and Huh7 cell lines. Results showed that the expression levels of AC012499.1, DRAIC, LINC01625, AF127577.4, AC068631.1, LINC01124, and AP003498.1 was highly expressed in Huh7 cell compared to L02 cell (Fig. 2J).

### Establishment of molecular types based on lncRNA expression

**Figure 3:**
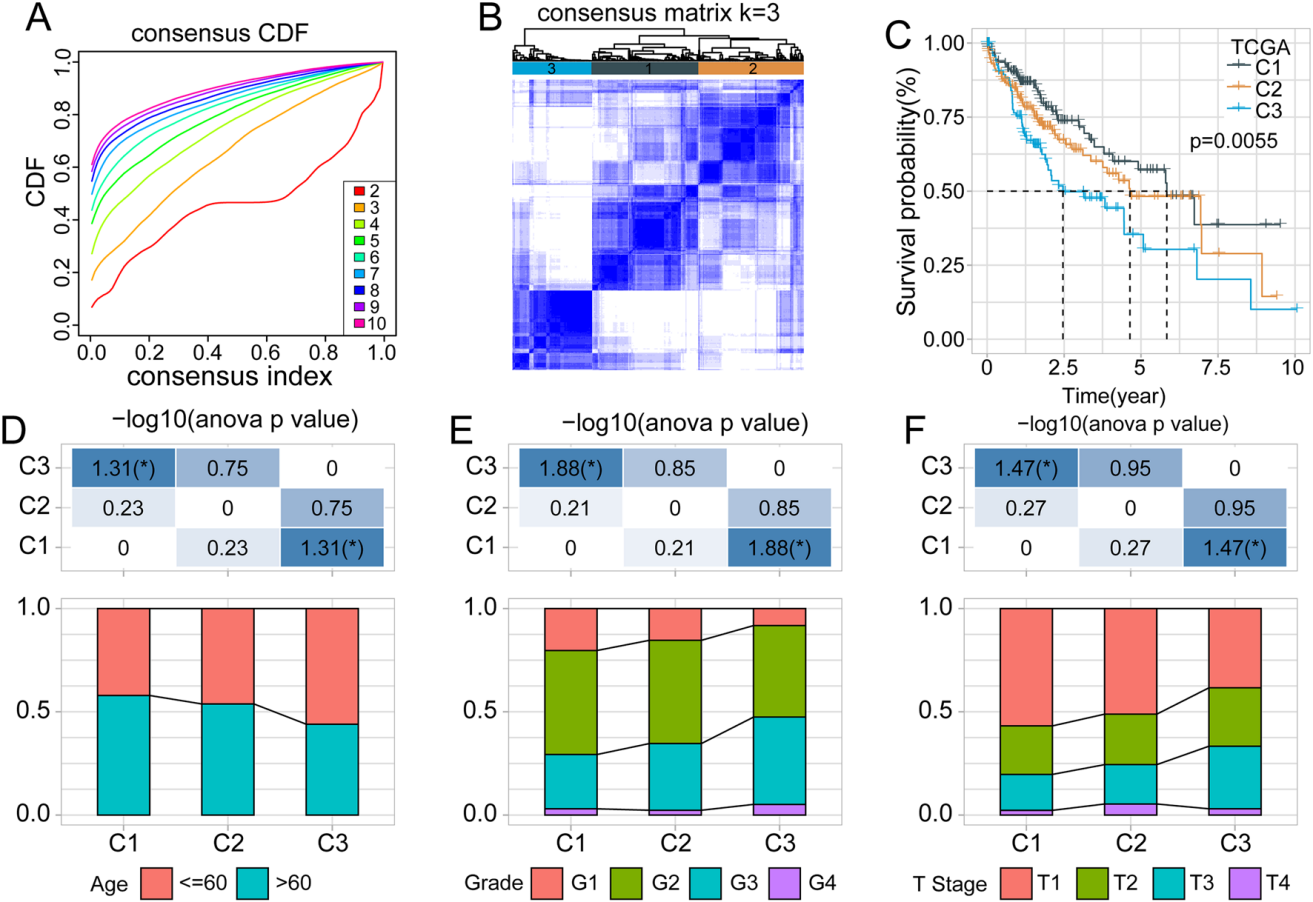
The fatty acid-associated long noncoding RNA (lncRNA) subtypes in the TCGA dataset and their clinical feature. (**A**) Cumulative distribution function (CDF) curves for the TCGA cohort samples. (**B**) Heatmap of the TCGA samples at consensus k = 3. (**C**) Prognostic overall survival (OS) curves for the fatty acid-associated lncRNA subtypes in the TCGA cohort. (**D**–**F**) Differences in the distributions of different clinical features across the molecular subtypes.

We used 743 lncRNAs from the combined TCGA and GSE76427 datasets to conduct univariate Cox regression analyses. Seven lncRNAs were confirmed to significantly correlate with prognosis (Supplementary Table 1), including *TRAF3IP2-AS1*, *SNHG10*, *AL157392.2*, *LINC02641*, *AL357079.1*, *AC046134.2*, and *A1BG-AS*. We clustered liver cancer samples in the TCGA and GSE76427 cohorts using ConsensusClusterPlus and obtained three molecular subtypes according to the cumulative distribution function (Fig. 3A,B). The prognostic features of these three subtypes showed significantly different. In the TCGA dataset, a better prognosis was associated with the C1 subtype than with the other subtypes, and patients with the C3 subtype had the shortest overall survival (OS) (Fig. 3C). A similar phenomenon was observed in the GSE76427 cohort (Supplementary Fig. 1D). These results suggested that the three subtypes defined by FA-associated lncRNA expression are consistent across diverse cohorts.

### Clinical and mutational characteristics of the molecular subtypes

**Figure 4:**
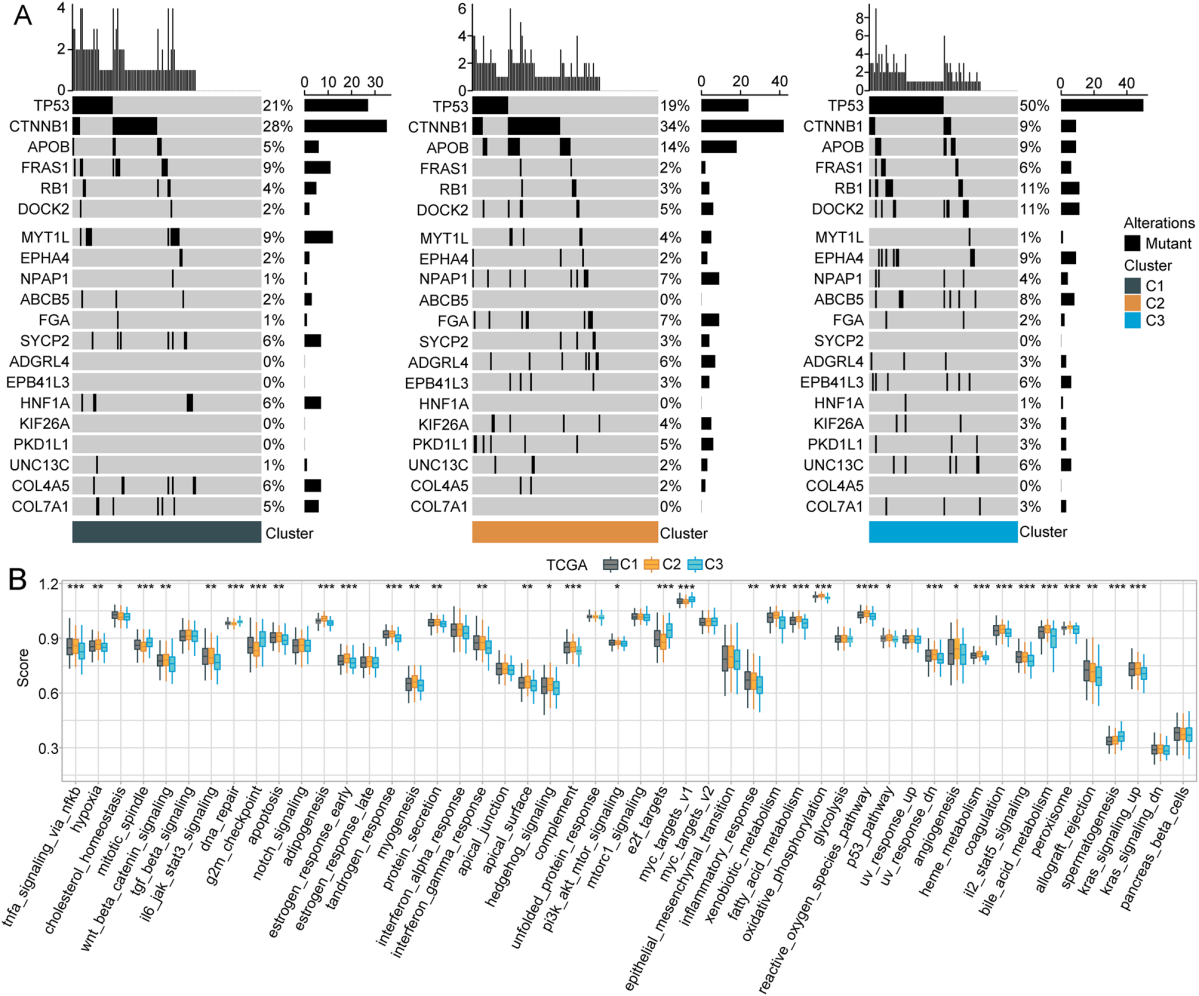
Mutational features of the defined molecular subtypes and differences in the hallmark signaling scores across three subtypes. (**A**) Differential somatic mutation analysis across the three molecular subtypes. (**B**) Boxplots showing tumor-related ssGSEA pathway scores in the TCGA dataset.

In the TCGA dataset, we compared the distribution of different clinical features across the three molecular subtypes, which revealed significant differences in age, tumor grade, and T stage between subtypes. Compared with C1 subtype, more patients older than 60 years were observed in C3 subtype (Fig. 3D). In addition, we found patients with C3 subtype might have worse tumor grade and T stage (Fig. 3E,F). The above results implied poorer prognosis for patients with C3 subtype. We examined the incidence of gene mutations in each subtype and found that some gene mutations were correlated with different prognostic outcomes for each subtype. The proportion of *TP53* mutations was significantly higher for the C3 subtype, which was associated with the worst prognosis, compared with the proportions in the C1 and C2 subtypes. By contrast, the proportion of *CTNNB1* mutants was significantly lower in the C3 subtype than in C1 and C2 (Fig. 4A).

### Hallmark signaling scores for each subtype

We used ssGSEA to score the samples from the TCGA and GSE76427 datasets and compared differences across subtypes. A total of 37 (74%) of 50 possible hallmark pathways in the TCGA data set were found to display significant differences across subtypes, including pathways associated with hypoxia, tumor necrosis factor (TNF)-α signaling via nuclear factor kappa B (NF-κB), and fatty-acid-metabolism signaling (Fig. 4B). In the GSE76427 dataset, 20 pathways with significant differences between subtypes were identified out of 50 possible pathways, containing mototic-spindle, xenobiotic-metabolism, oxidative-phosphorylation, fatty-acid-metabolism, bile-acid- metabolism signaling (Supplementary Fig. 1E). We could recognize that fatty-acid-metabolism pathway was hallmark signaling in three subtypes and presented observable difference.

### Immunological infiltration associated with each molecular subtype

**Figure 5:**
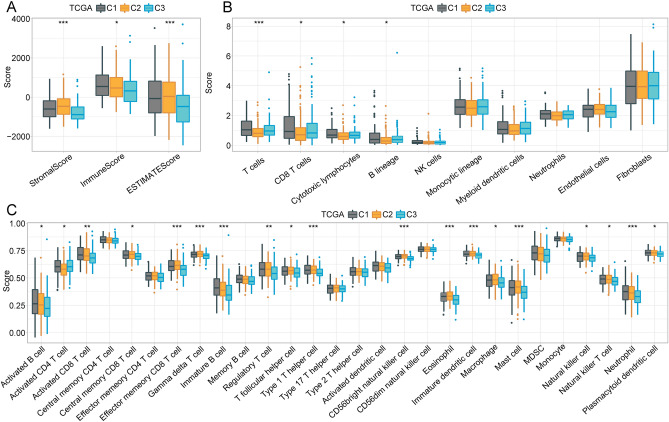
Immune features across the three subtypes in the TCGA datasets. (**A**) Immune microenvironment scores across subtypes (TCGA). (**B**) Differential infiltration of immune cells in three subtypes (TCGA). (**C**) Differences in scores for immune-related pathways across the three subtypes.

To further clarify differences in the immune microenvironments associated with molecular subtypes defined by the expression of FA-associated lncRNAs, we evaluated the immune cell infiltration of patients in two HCC cohorts based on gene expression levels. A variety of immunocyte marker genes were identified in the literature^21^, and ESTIMATE and MCP-Counter software were used to assess the immunochemical environment. Differences in immune cell distributions were observed among the molecular subtypes defined according to FA-associated lncRNA expression. In TCGA cohorts, three subtypes in Stromal score, Immune score and ESTIMATE score embodied remarkable differences, and the C3 subtype, which was associated with poor prognosis, displayed lower scores (Fig. 5A). The three molecular subtypes displayed significant differences in part immune cell types, such as T cells, CD8 T cell, Cytotoxic lymphocytes and B lineage cells (Fig. 5B). Several immune-related pathways were found to be downregulated in the C3 subtype, whereas the prognosis associated with the immunophenotype associated with the C1 subtype was improved, according to the ESTIMATE score (Fig. 5C). In GSE76427 cohorts, it was observed parallel findings. These results suggest that the subtypes displayed stable and consistent molecular characteristics (Supplementary Fig. 2).

**Figure 6:**
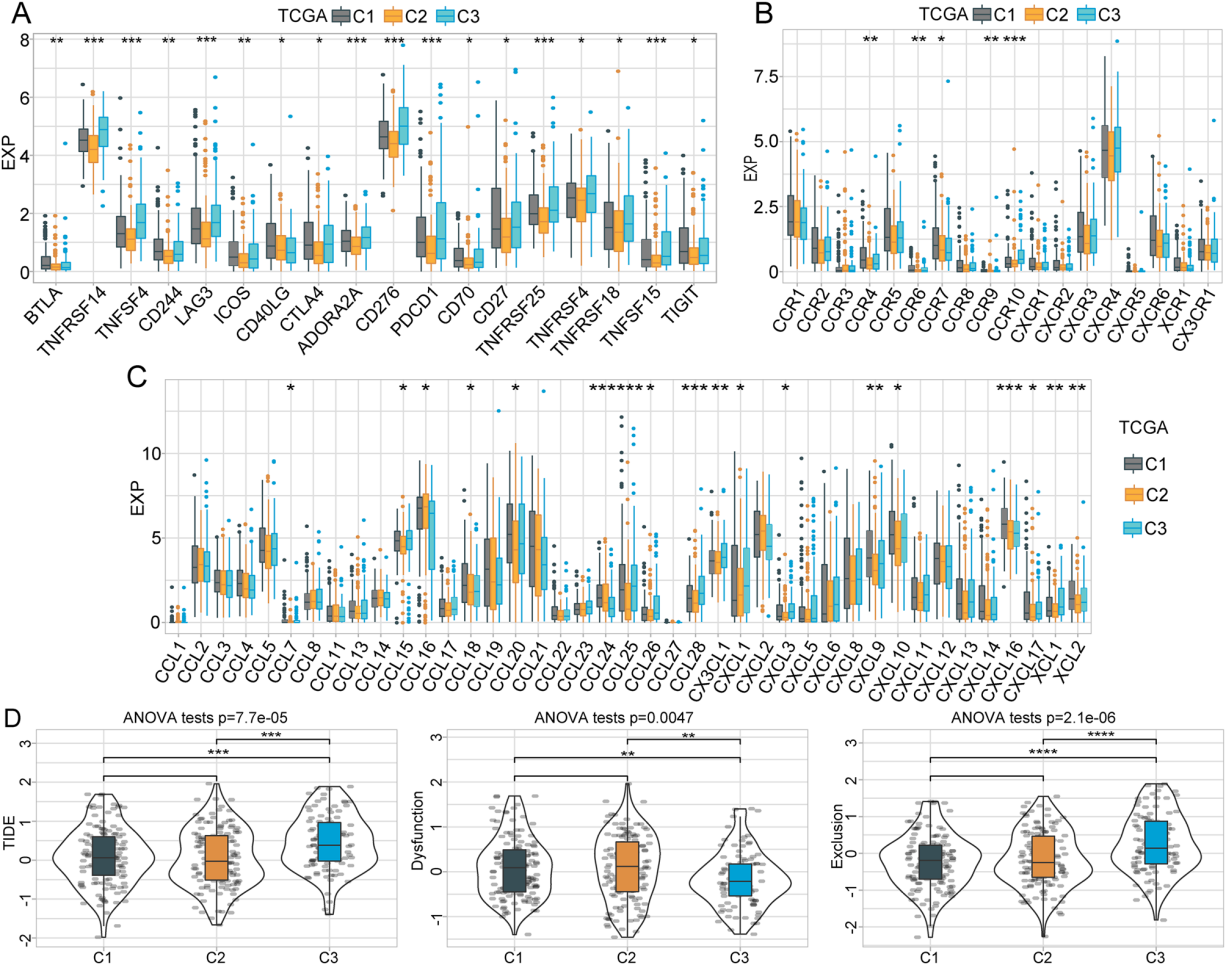
Immunotherapy differences in molecular subtypes. (**A**) Differential distribution of immune checkpoint expression across subtypes from the TCGA. (**B**,**C**) Differences in chemokine and chemokine receptor expression across subtypes in the TCGA. (**D**) Differences in TIDE and CAF scores and survival for TCGA immunotherapy groups.

These combined analyses showed that the C3 subtype was associated with the worst prognosis and displayed the lowest immune score among all three subtypes. To explore why the C3 subtype had the worst prognosis and lowest immune scores, we assessed the distribution of immune checkpoints in all three subtypes. In the TCGA dataset, 18 (38.30%) of 47 immune checkpoints was presented in significant differences between subtypes, with most presenting high expression in the C3 subtype associated with poor prognosis (Fig. 6A). By contrast, 15 (33.33%) of the 45 immune checkpoints identified in the GSE76427 dataset were significantly different, most of which showed downregulated expression in the C3 subtype (Supplementary Fig. 3A).

Chemokines play key roles in tumorigenesis and cancer development, attracting multiple immune cells to the tumor microenvironment. Chemokines are thought to assist T cell entry into tumors, influencing the tumor response to the immune system and therapy. Thus, we analyzed whether chemokines differentially expressed across these three subtypes. We calculated differences in chemokine gene expression in the TCGA cohort, as shown in Fig. 6C, which revealed that 18 of 41 chemokines (43.90%) were differentially expressed across subtypes. Similarly, in the GSE76427 cohort, we identified 10 (25%) chemokines with significant differences across the three subtypes (Supplementary Fig. 3C). These data suggest that the degree of immune cell infiltration may vary across different metabolic subtypes, which may contribute to observed differences in tumor progression and immunotherapy efficacy. We also compared the expression of chemokine receptor genes between the different metabolic subtypes. As shown in Fig. 6B, in the TCGA data set, 5 of 18 (27.78%) chemokine receptor genes showed significant differential expression across metabolic subtypes. In the GSE76427 dataset, 5 of 17 chemokine receptor genes displayed significant differential expression across metabolic subtypes (Supplementary Fig. 3B).

### TIDE analysis

We used the TIDE to evaluate the potential clinical response to immunotherapy in the three molecular subtypes defined using the TCGA dataset. A higher TIDE predictive score indicated a higher probability of immune escape, suggesting that patients were less likely to benefit from immunotherapy. As shown in Fig. 6D, the TIDE score for the C3 subtype was significantly higher than the TIDE scores for the C1 and C2 subtypes in the TCGA cohort, suggesting a higher likelihood of immune escape in the C3 subtype and a likely unsatisfactory response to immunotherapy. Compared with the C3 subtype, the dysfunction scores for the C1 and C2 subtypes were higher, whereas the exclusion scores of the C1 and C2 subtypes were lower (Fig. 6D). We also observed similar results when TIDE analysis was applied to the GSE76427 dataset (Supplementary Fig. 3D).

### Functional enrichment analysis of the molecular subtypes

**Figure 7:**
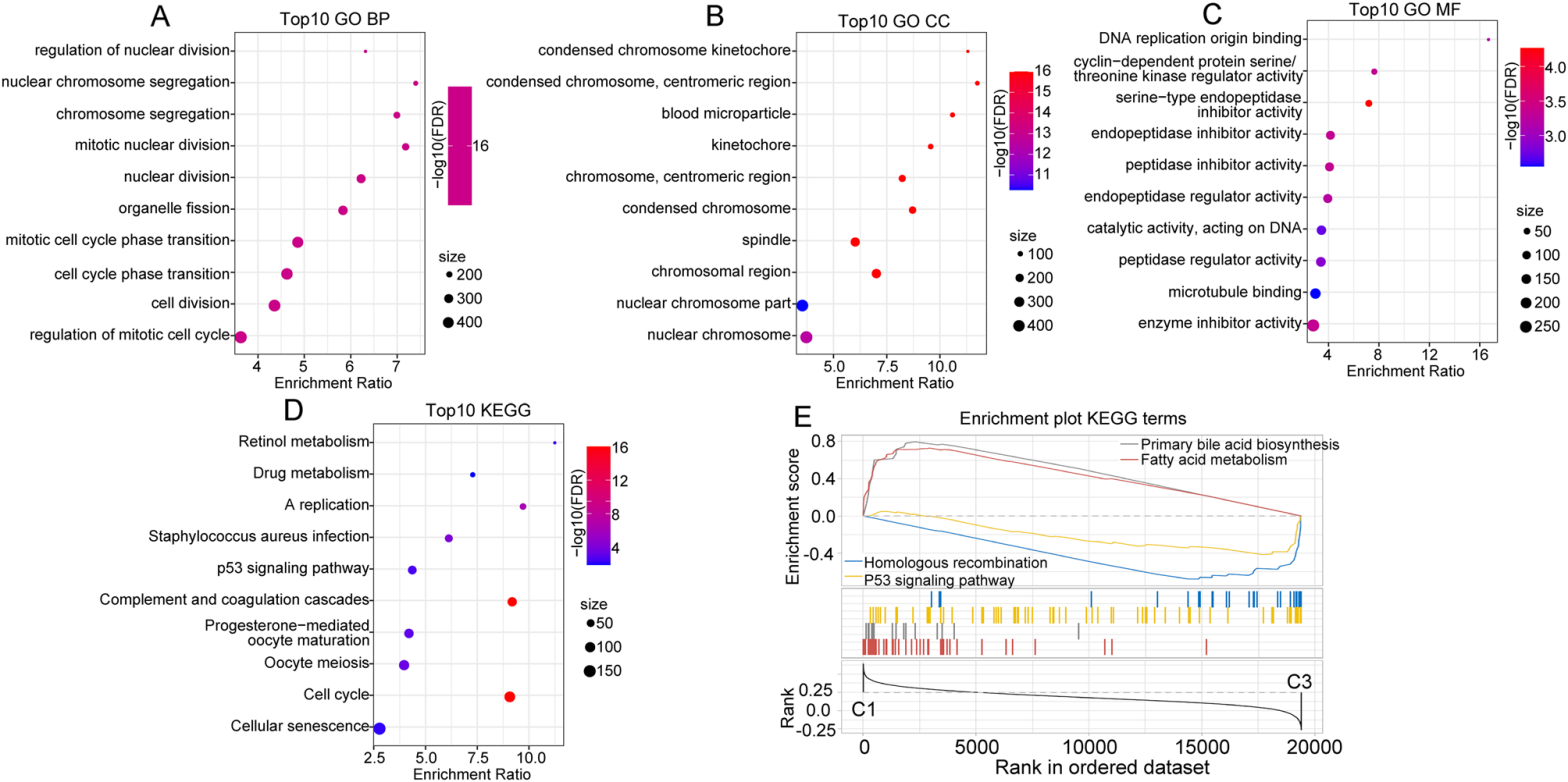
Functional enrichment of fatty acid-related long noncoding RNAs (lncRNAs). (**A**–**D**) Gene Ontology (GO) and Kyoto Encyclopedia of Genes and Genomes (KEGG) analysis of differentially expressed genes (DEGs) from the TCGA dataset. (**E**) Gene set enrichment analysis (GSEA) of the C1 and C3 subtypes (TCGA).

In exploration of clinical feature, we could find that significant difference from C3 and C1 subtype were testified. To verify whether functional differences exist between our defined molecular subtypes, we compared the C3 and C1 subtypes using “limma” to identify differentially expressed genes (DEGs). In the TCGA cohort, 557 DEGs were identified, including 387 genes that were upregulated in C3 and 170 genes that were downregulated in C3 relative to C1. In the GSE76427 dataset, 564 DEGs were defined, including 309 genes upregulated in C3 and 255 genes downregulated in C3 relative to C1. We conducted KEGG pathway analysis and Gene Ontology (GO) functional enrichment analysis on identified DEGs via the R package WebGestaltR. In the GO analysis of the TCGA database, 266 pathways were associated with biological processes (BP), 28 pathways were associated with molecular functions (MF), and 60 pathways were associated with cellular components (CC), and we selected the top 10 pathways for each analysis (Fig. 7A–C). KEGG pathway enrichment analysis identified 13 significantly represented signaling pathways. Partial annotation results demonstrated that DNA replication, cell cycle, and p53 signaling pathways, as well as tumor and immune-related pathways, were strongly correlated with the identified DEGs (Fig. 7D). In addition, metabolism-related pathways, including drug metabolism and retinol metabolism, were significantly enriched.

The GO functional annotation of DEGs in the GSE76427 dataset revealed 119 BP pathways, 31 MF pathways, and 13 CC pathways. KEGG enrichment analysis revealed 21 significant pathways, including several pathways related to cancer, such as cell cycle and microRNAs. Metabolism-related pathways, including drug metabolism, metabolism of xenobiotics by cytochrome P450, and FA elongation, were also significantly enriched (Supplementary Fig. 4A–D).

To further investigate the biological pathways associated with the different molecular subtypes, we used GSEA for pathway analysis, comparing the C1 and C3 subtypes using all candidate gene sets included in the KEGG database. Pathways related to metabolism, such as primary blue acid biosynthesis and FA metabolism, were significantly enriched in the C1 subtype relative to the C3 subtype in the TCGA dataset. In addition, pathways such as homologous recombination and P53 signaling were associated with the C3 subtype (Fig. 7E). The enrichment results in the GSE76427 dataset were consistent with the results in the TCGA dataset (Supplementary Fig. 4E), and tumor-associated pathways were remarkably enriched in the C2 subtype.

### Comparison with existing molecular subtypes

**Figure 8:**
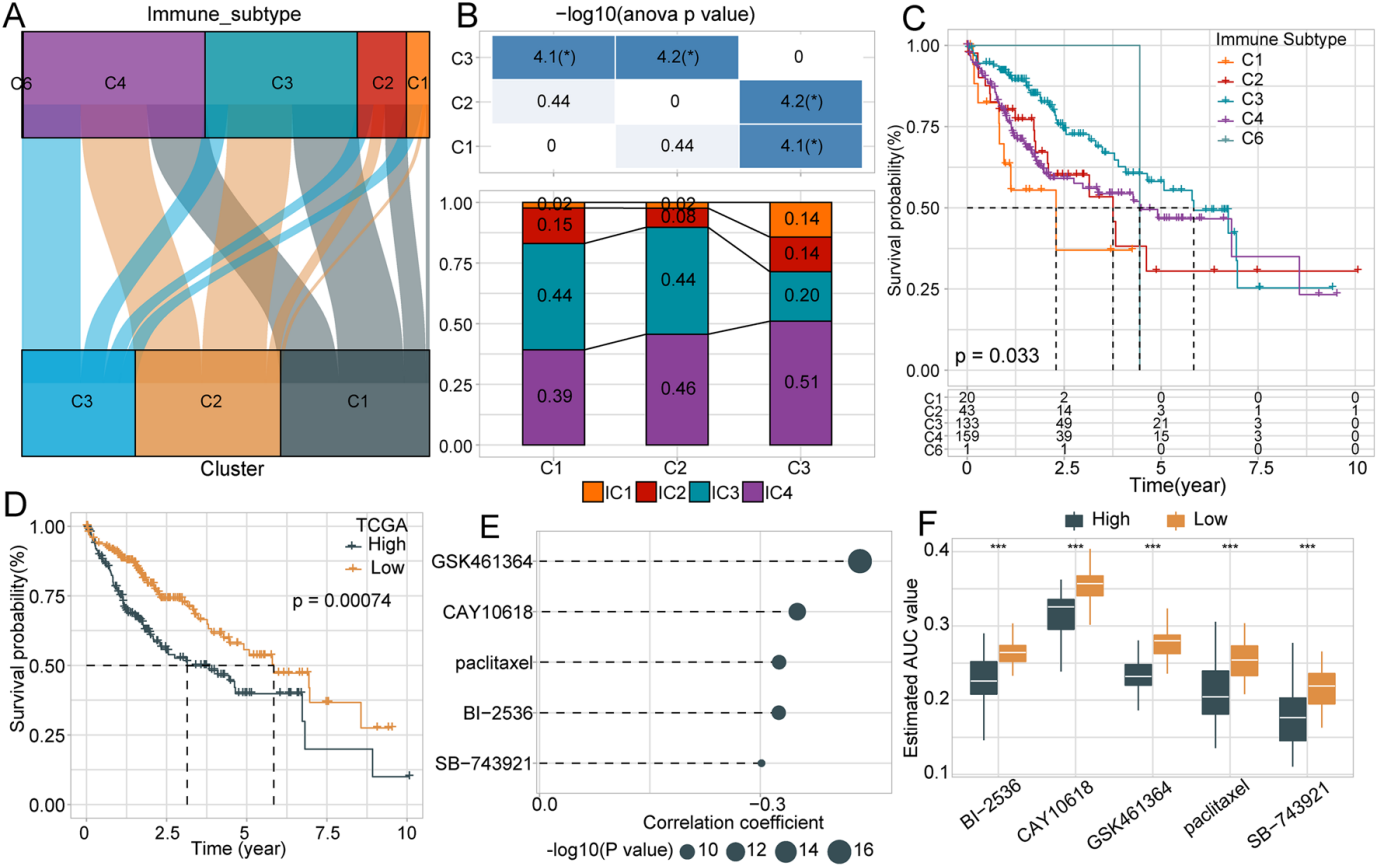
Comparison between existing subtypes and our molecular subtypes and drug sensitivity analysis. (**A**) Sanki diagram comparing our molecular subtypes and existing molecular subtypes. (**B**) Comparison immune subtype distributions across our three defined molecular subtypes. (**C**) Survival curves associated with existing immune subtypes. (**D**) Kaplan–Meier (KM) curves for high- and low-risk groups in the TCGA cohort. (**E**) Drug sensitivity to chemotherapy in patients from the TCGA dataset based on the CTRP2.0 database. (**F**) Drug sensitivity to chemotherapy in patients from the TCGA dataset based on the PRISM database.

The six immune infiltration subtypes identified in human tumors include C1 (wound-healing), C2 (interferon-γ-dominant), C3 (inflammation), C4 (lymphocyte depletion), C5 (immunologically silenced), and C6 (transforming growth factor-β-dominant). Studies have shown that existing C1, C2, and C6 are associated with poor prognosis. Most LIHC patients in the TCGA-LIHC database are categorized as presenting with existing C3 and C4 immune subtypes, whereas existing C5 immune subtype was not detected in any sample in the HCC TCGA dataset. Survival curve analysis showed significant differences in OS among these previously defined subtypes, revealing poor prognosis associated with existing C1, C2, and C4 (Fig. 8C). We further compared the sample distribution between our molecular subtypes and existing subtypes, which revealed that our C3 molecular subtype, which was associated with poor prognosis, contained a higher proportion of immune subtypes associated with poor prognosis, including existing C1, C2, and C4. Our molecular subtypes C1 and C2, which were associated with better prognosis and presented in larger proportions of existing C3 immune subtype (Fig. 8A,B).

### Potential drug therapy analysis

Based on the expression profiles of the seven identified lncRNAs, we conducted a multivariate Cox analysis using the TCGA dataset to obtain correlation coefficients. Samples were divided into high- and low-risk groups relative to the median risk value. We also validated the expression of these seven lncRNAs in the GSE76427 dataset using multivariate Cox regression. Prognosis was significantly different between the high- and low-risk groups for both HCC datasets, indicating that these seven lncRNAs might be useful for predicting prognosis in HCC patients. We analyzed the drug sensitivity associated with these lncRNAs by comparing the high-risk and low-risk groups. Compared with the low-risk group, we found that the high-risk group was more sensitive to five drugs in the CTRP2.0 database, including BI 2536, CAY10618, GSK461364, paclitaxel, and SB 743921 (Fig. 8D–F). Analogously, the high-risk group showed high sensitivity to epothilone-b, ispinesib, LY2606368, and YM 155 in the PRISM database (Supplementary Fig. 5A–C). These results broaden new perspectives for drug targets for personalized treatment of HCC.

## Discussion

Emerging evidence indicates that lncRNAs function as promoters or suppressors that affect the occurrence and progression of cancer^10,22^. However, lncRNAs do not act alone, and the contributions of other signaling factors are indispensable. FA is a significant component of the lipid metabolic process and can be synthesized or converted into complex lipid species, participating in the activation of various signaling cascades. The meditation effects of lncRNAs in FA signaling pathways have been slowly revealed in diverse tumor types^23–25^. However, there is not much corresponding study regarding association between FA and lncRNAs in HCC.

We obtained samples from the TCGA, HCCDB18, and GSE14520 databases and identified significant hallmark pathways in HCC by performing ssGSEA and Cox regression analyses, revealing the FA pathway as an important hallmark pathway in HCC. The current investigation confirmed that long-chain acyl CoA synthetase 4 (ACSL4) participates in FA pathways to advance the progression of HCC, resulting in abnormal lipid metabolism^26^. We identified 743 lncRNAs associated with FA signaling in both the TCGA and GSE764277 datasets and selected the top seven lncRNAs strongly correlated with prognosis, including *TRAF3IP2-AS1*, *SNHG10*, *AL157392.2*, *LINC02641*, *AL357079.1*, *AC046134.2*. and *A1BG-AS*, some of which have previously been reported to be involved in the pathogenesis of various cancers. Compared with normal cells, *TRAF3IP2-AS1* was found to be expressed at low levels in response to the upregulation of the NONO-TFE3 fusion protein in NONO-TFE3 translocation renal cell carcinoma (tRCC). The downregulation of *TRAF3IP2-AS1* resulted in reduced *PTEN* expression, facilitating the progression of *NONO-TFE3* tRCC by inducing the m6A modification of *PARP1* mRNA. *SNHG10* is expressed at higher levels in prostate cancer tissue than in normal tissue, contributing to a shorter survival time among patients. Thus, screening for these lncRNAs may provide additional information regarding the underlying molecular mechanism involved in HCC development and progression.

We used ConsensusClusterPlus to examine the samples obtained from the TCGA and GSE764277 cohorts, which defined three molecular subtypes: C1, C2, and C3. These three subtypes were associated with differential prognosis. To verify and understand the mechanisms contributing to prognostic differentiation among these three subtypes, we focused on differences in mutational features, comparisons with existing molecular subtypes, and identifying functional enrichment pathways. The occurrence of *TP53* mutations was observed with a higher probability in our C3 subtype, which is associated with the worst prognosis among the three subtypes defined in this study. *TP53* plays important roles in cell cycle arrest, apoptosis, metabolism, DNA repair, and resistance to chemotherapy and has been shown to mutate frequently in malignant tumors^27^, and *TP53* mutations are detected in nearly 30% of all BC cases^28^. Our defined C3 subtype was also associated with the previously defined C1, C2, and C4 immune infiltration subtypes, which were associated with poor prognosis in patients. The high probability of *TP53* mutations and the presence of existing immune subtypes associated with poor prognosis support and explain the poor prognosis observed for our C3 subtype.

The GO and KEGG analyses identified DNA replication and cell cycle pathways as being strongly associated with DEGs identified in the comparison between our C3 and C1 subtypes. DNA replication is inactive in most differentiated cells, and DNA replication proteins are typically expressed at low levels^29^. However, cancer cells are known to present with highly activated DNA replication processes. In BC stem-like cells, mini-chromosome maintenance protein 10 (MCM10) dramatically promotes DNA replication, compensating for DNA replication stress associated with c-Myc induction^30^. The cell cycle describes the vital biological process that controls the duplication of genetic materials, cell division, and growth^31^. Cell cycle dysfunction is commonly associated with disease occurrence and contributes to the rapid proliferation and apoptosis properties observed in multiple cancer types. The reduced activation of the transcription factor friend leukemia integration 1 (FLI1) could negatively affect the expression of cyclin D1 (CCND1) and E2F transcription factor 2 (E2F2), resulting in the arrest of the cell cycle at the G1/S phase and facilitating the progression of non-neoplastic lung cells^32^. Therefore, we assume that these DEGs might participate in tumor-related pathways, including DNA replication and the cell cycle, contributing to shorter survival times in the C3 subtype.

Immunotherapy has become increasingly popular for the treatment of numerous diseases; however, conventional immunotherapy is challenging for liver diseases due to the specific immune response tolerance of the liver^33^. Therefore, we used the ESTIMATE algorithm and MCP-Counter software to estimate the immune scores of our three defined subtypes. The immune scores of the C3 subtype were lower than those for the C1 and C2 subtypes. Immune checkpoint analysis revealed that 38.30% (TCGA dataset) and 33.33% of immune checkpoints (GSE764277 dataset) were differentially expressed across the three molecular subtypes. However, different immune checkpoints were identified as differentially expressed in the C3 subtype between these two datasets.

Chemokines refer to small cytokines and signaling proteins secreted by diverse cells that are involved in enhancing the antitumor response of the tumor microenvironment and have been shown to be beneficial for cancer patients^34^. The upregulation of the chemokines CXCL10 and CXCR3 have been positively correlated with satisfactory outcomes in patients with HCC^35^. Interferon regulatory factor 1 (IRF-1) induces HCC apoptosis through CXCL10/CXCR3 signaling^36^. To understand immunotherapy efficiency in our defined C1, C2, and C3 subsets, we also analyzed the expression of chemokines and chemokine receptor genes and found remarkable differences in expression profiles across these three subtypes, which might provide clearer guidance for the application of immune targeted therapy in HCC patients. The seven identified lncRNAs were used to construct novel predictive prognostic models for HCC patients.

Our study has some limitations. The validation and elucidation of the complex mechanisms underlying differences in prognosis between these three subtypes require additional experiments. The specific associations between the expression profiles of the identified lncRNAs and survival time among HCC patients also require further exploration.

In conclusion, we stratified two HCC cohorts according to key lncRNAs associated with FA signaling pathways and defined C1, C2, and C3 subtypes with significant differences in prognosis. This classification method has been further verified in the clinical characteristics, mutation feature, and immune microenviroment, and made a more comprehensive classification of patients with hepatocellular carcinoma, which was conducive to the personalized diagnosis and treatment of patients. The poor prognostic outcomes associated with our C3 subtype might be robustly associated with alterations in the DNA replication and cell cycle pathways, indicating the need for further studies examining the involvement of FA-associated lncRNAs in these processes. In addition, the seven identified lncRNAs (*TRAF3IP2-AS1*, *SNHG10*, *AL157392.2*, *LINC02641*, *AL357079.1*, *AC046134.2*, and *A1BG-AS*) might guide the further exploration of prognostic biomarkers in HCC.

## Materials and methods

### Data collection and data preprocessing

RNA sequence data and clinical information for patients with liver cancer (LIHC) were obtained from TGCA, and the data were processed as follows. First, samples without survival information were removed, and tumor samples were retained. Next, Ensembl gene IDs were matched with GeneSymbol. We downloaded the ICGC-LIRI-JP datasets from the HCCDB website (http://lifeome.net/Database/hccdb/home.html). The GSE76427 and GSE14520 data sets were downloaded from the GEO database (https://www.ncbi.nlm.nih.gov/geo/). Samples with living time and survival status were retained and SEQMAP was used to re-annotate genes in two datasets. We obtained the gene transfer format files for the V32 annotation from the GENCODE website (https://www.gencodegenes.org/), which we used to divide the TCGA expression profiles and GSE76427 data into mRNA and lncRNA. lncRNA expression data shared by the TCGA data and the GSE76427 data were retained. We also accessed gene sets from the H.all.v7.4.symbols.gmt file from the gene set enrichment analysis (GSEA) website^37^.

### Identification of significant hallmark pathways in HCC

We utilized the single-sample GSEA (ssGSEA) method to calculate hallmark pathway scores for samples included in the TCGA, HCCDB18, and GSE14520 datasets. The hallmark pathways related to HCC prognosis were identified by univariate Cox analysis based on the scores obtained for each data set individually, and the intersection of important hallmark pathways identified in all three data sets was used to define shared hallmark pathways.

### Screening lncRNAs associated with FA signaling

To validate effective lncRNAs involved in FA signaling, we adopted an integrated pipeline based on the processes described by several studies^38,39^. Briefly, we used correlations between mRNA and lncRNA expression levels to estimate target mRNA, which were ranked in descending order. The “fgsea” R package was utilized to analyze the ordered gene list and identify whether lncRNAs related to FA pathways were enriched at the top or bottom of gene lists. After estimating the total enrichment score (TES) for FA signaling from the entire lncRNA population, several lncRNAs with significant TES values were considered to be FA-associated lncRNAs, in accordance with the permutation test framework. In expression matrixes for lncRNA and mRNA, an lncRNA i and an mRNA j detected in n patients were expressed as LNC(i) = (lnc_1_, lnc_2_, …, lnc_n_) and M(j) = (m_1_, m_2_, …, m_n_), respectively. We used the ESTIMATE R package to quantify tumor purity in n patients, defined as P = (p_1_, p_2_, …, p_n_). The first-order partial correlation coefficient (PCC) between an lncRNA i and an mRNA j was calculated by eliminating the effects of tumor purity:

$$\mathrm{PCC}(\mathrm{ij})=\frac{\mathrm{Rlncm}-\mathrm{Rlncp}\times \mathrm{Rmp}}{\sqrt{1-{\mathrm{R}}^{2}\mathrm{lncp}}\times \sqrt{1-{\mathrm{R}}^{2}\mathrm{mp}}}$$

where Rlncm, Rlncp, and Rmp are the respective Pearson’s correlation coefficients between an lncRNA i and an mRNA j, between an lncRNA i and tumor purity p, and between an mRNA j and tumor purity p. Subsequently, the P-value for PCC (ij), defined as P(ij), was measured as follows:

$$\mathrm{P}\left(\mathrm{ij}\right)=2\times \mathrm{pnorm}(-\left|\mathrm{PCCij}\times \sqrt{\frac{\mathrm{n}-3}{1-{\mathrm{PCC}}^{2}\mathrm{ij}}}\right|)$$

where Pnorm is the normal distribution function, and n is the number of samples. For an lncRNA i, the rank index (RI) for an mRNA j was calculated as:

$$ {\text{RI }}\left( {{\text{ij}}} \right) = \, - {\text{ln}}\left( {{\text{P}}\left( {{\text{ij}}} \right) \, \times {\text{ sign }}\left( {{\text{PCC }}\left( {{\text{ij}}} \right)} \right)} \right) $$

The sign function is a mathematical function for extracting the signs for PCC (ij). All mRNAs were sorted in descending RI order to perform GSEA. The genes associated with FA signaling are presented as an ordered gene list. For an lncRNA i, the enrichment score and P-value (adjusted by the false-discovery rate [FDR]) were assessed using the “fgsea” R package and then combined into a TES:

$$ {\text{TES}}\left( {\text{i}} \right) \, = \, \left( {{1} - {\text{2Pi}}} \right) \, \times {\text{ sign }}\left( {{\text{ESi}}} \right) $$

Therefore, TES values ranged from − 1 to 1, and lncRNAs with an absolute TES value > 0.95 and an FDR < 0.05 were validated as FA-associated lncRNAs.

### Validation of lncRNA subtypes related to FA

Expressed lncRNAs associated with FA pathways were input into ConsensusClusterPlus^40^ to construct a consistency matrix for classifying TCGA and GSE76427 samples into subtypes^41^. The Kaplan–Meier (KM) algorithm and maximum distance were used to measure distance and perform 500 Bootstraps. Each bootstrap process included 80% of the data as a training set. We selected the number of clusters as 2 to 10 and calculated the consistency matrix and the consistency cumulative distribution function to determine the best classification method.

### GSEA and functional annotation

To explore biological process pathways associated with various molecular subtypes, we used the Kyoto Encyclopedia of Genes and Genomes (KEGG)^21^ database to perform GSEA. In addition, we adopted the WebGestaltR (v0.4.4) software package to present functional annotations of different genes.

### Tumor immune microenvironment analysis

We evaluated the immune infiltration scores of samples using ESTIMATE, MCP-Counter, and ssGSEA and compared differential distributions across different subtypes. Tumor Immune Dysfunction and Exclusion (TIDE http://tide.dfci.harvard.edu/), a computational framework, was used to estimate the ability of tumor immune escape from the gene expression profiles of cancers samples.

### Construction of risk models based on lncRNA expression profiles

To estimate the prognostic abilities of critical lncRNAs, multivariate regression analysis was conducted on samples from the TCGA dataset, which resulted in a correlation coefficient for each lncRNA. The following formula was used to calculate the risk score for each patient: risk score = (βi × EXPi), where i represents the expression levels of lncRNAs associated with FA, and β is the coefficient of the gene for the corresponding lncRNA based on univariate Cox regression. Patients were divided into high- and low-risk groups according to the median risk score. The KM method was used to generate a survival curve for prognosis, and a quota test was used to determine significant differences.

### Analysis of drug sensitivity

Chemotherapeutic sensitivity was predicted for the high- and low-risk groups using the CTRP2.0 and PRISM databases, which contain sensitivity data for 481 and 1448 compounds, respectively. Area under the receiver operating characteristic curve (AUC) values were determined to measure drug sensitivity in these two datasets, with a lower AUC value indicating enhanced sensitivity to treatment^42^. Cell line expression profiles obtained from the Cancer Cell Line Encyclopedia database were used as the training set for predicting drug sensitivity, and TCGA-LIHC was used as the test set.

### Cell culture and quantitative reverse transcription PCR (qRT-PCR)

We obtained L02 and Huh7 cell lines from Chinese Academy of Sciences (Shanghai, China). These cells were cultured in DMEM (Gibco) supplemented with 10% FBS (Gibco) 10% fetal bovine serum at 37 °C under 5% CO_2_ in a humidified incubator. Total RNA was isolated using TRIzol reagent (Invitrogen). A HiScript II QRT SuperMix Kit (Vazyme, China) was used to synthesize the cDNA. A ChamQ SYBR qPCR Master Mix (Vazyme, China) was used for real-time PCR with a Light Cycler 96 detection system (Roche). The information of primers sequences can be found in supplementary Table 2.

## Supplementary Information

Supplementary Legends.

Supplementary Figure 1.

Supplementary Figure 2.

Supplementary Figure 3.

Supplementary Figure 4.

Supplementary Figure 5.

Supplementary Tables.

## Data Availability

The datasets used and analyzed during the current study available from the corresponding author on request.
