# Supplementary material for: A novel classification of HCC basing on fatty-acid-associated lncRNA
Source: Sci Rep. 2022 Nov 7;12:18863. doi: 10.1038/s41598-022-23681-0 (PMC9640627; doi:10.1038/s41598-022-23681-0)
Supplement: Supplementary file 1 — Supplementary Legends. [file 41598_2022_23681_MOESM1_ESM.docx]

**Supplementary Figure Legend**

**Supplementary Fig. 1** (A) Wayne diagram of fatty acid–related long noncoding RNAs (lncRNAs) in the TCGA and GSE76427 datasets. (B) Cumulative distribution function (CDF) curves for the GSE76427 cohort samples. (C) Heatmap of the GSE76427 samples at consensus k = 3. (D) Prognostic overall survival curves for the fatty acid–associated lncRNA subtypes in the GSE76427 cohort. (E) Boxplots showing tumor-related ssGSEA pathway scores in the GSE76427 dataset.

**Supplementary Fig. 2** (A) Immune microenvironment scores across subtypes (GSE76427). (B) Differential infiltration of immune cells in three subtypes (GSE76427). (C) immune-related pathways in three subtypes (GSE76427).

**Supplementary Fig. 3** (A) Differential distribution of immune checkpoint expression across subtypes from the GSE76427. (B-C) Differences in chemokine and chemokine receptor expression across subtypes in the GSE76427. (D) Differences in TIDE and CAF scores and survival for TCGA immunotherapy groups.

**Supplementary Fig. 4** (A-D) Gene Ontology (GO) and Kyoto Encyclopedia of Genes and Genomes (KEGG) analysis of differentially expressed genes (DEGs) from the GSE76427 dataset. (E) Gene set enrichment analysis (GSEA) of the C1 and C3 subtypes (GSE76427).

**Supplementary Fig. 5** (A) Kaplan–Meier (KM) curves for high- and low-risk groups in the GSE76427 cohort. (B) Drug sensitivity to chemotherapy in patients from the GSE76427 dataset based on the CTRP2.0 database. (C) Drug sensitivity to chemotherapy in patients from the GSE76427 dataset based on the PRISM database.
