# Supplementary figures and images for: A novel classification of HCC basing on fatty-acid-associated lncRNA
Source: Sci Rep. 2022 Nov 7;12:18863. doi: 10.1038/s41598-022-23681-0 (PMC9640627; doi:10.1038/s41598-022-23681-0)

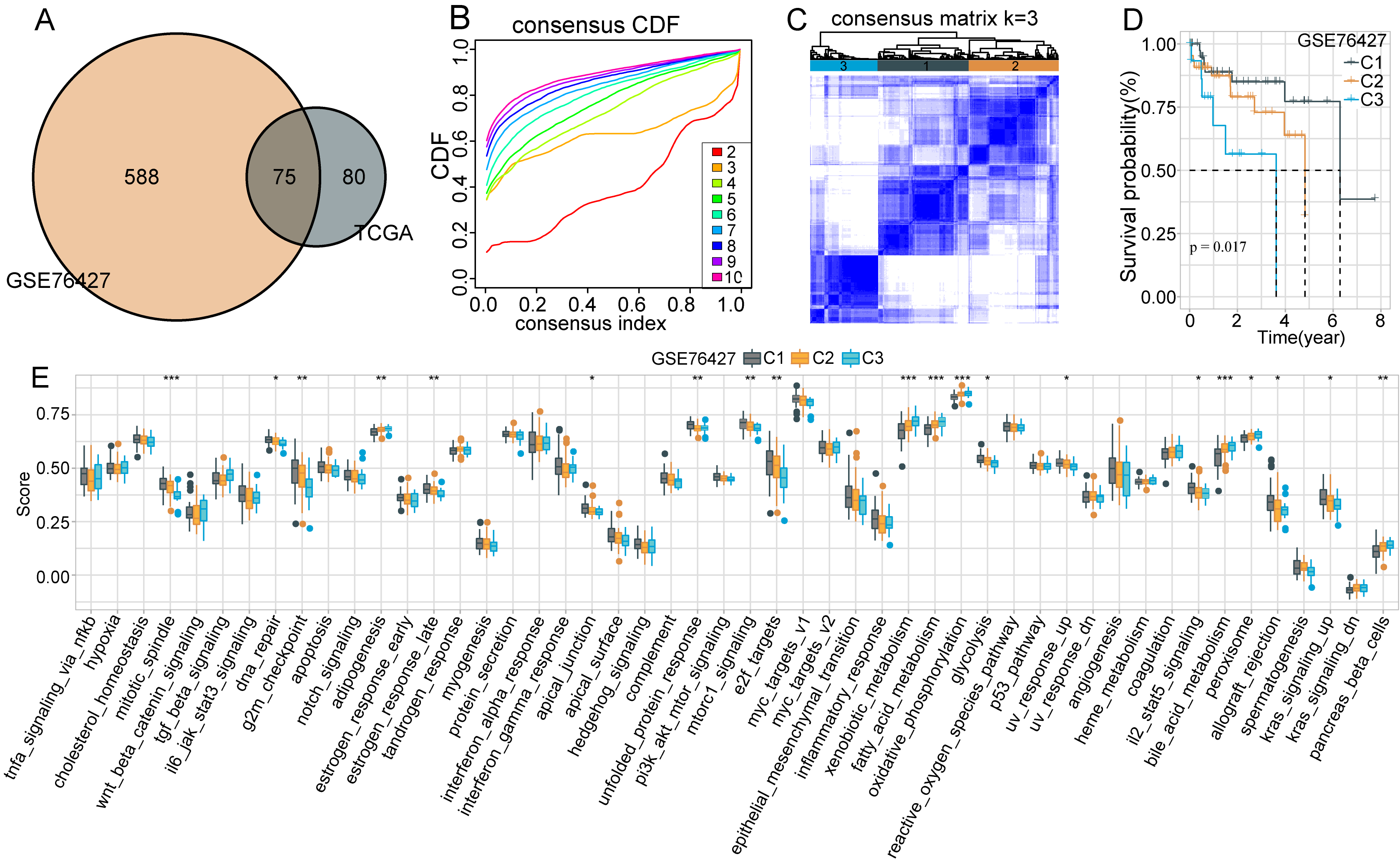

Supplement: Supplementary file 2 — Supplementary Figure 1. [file 41598_2022_23681_MOESM2_ESM.tif]

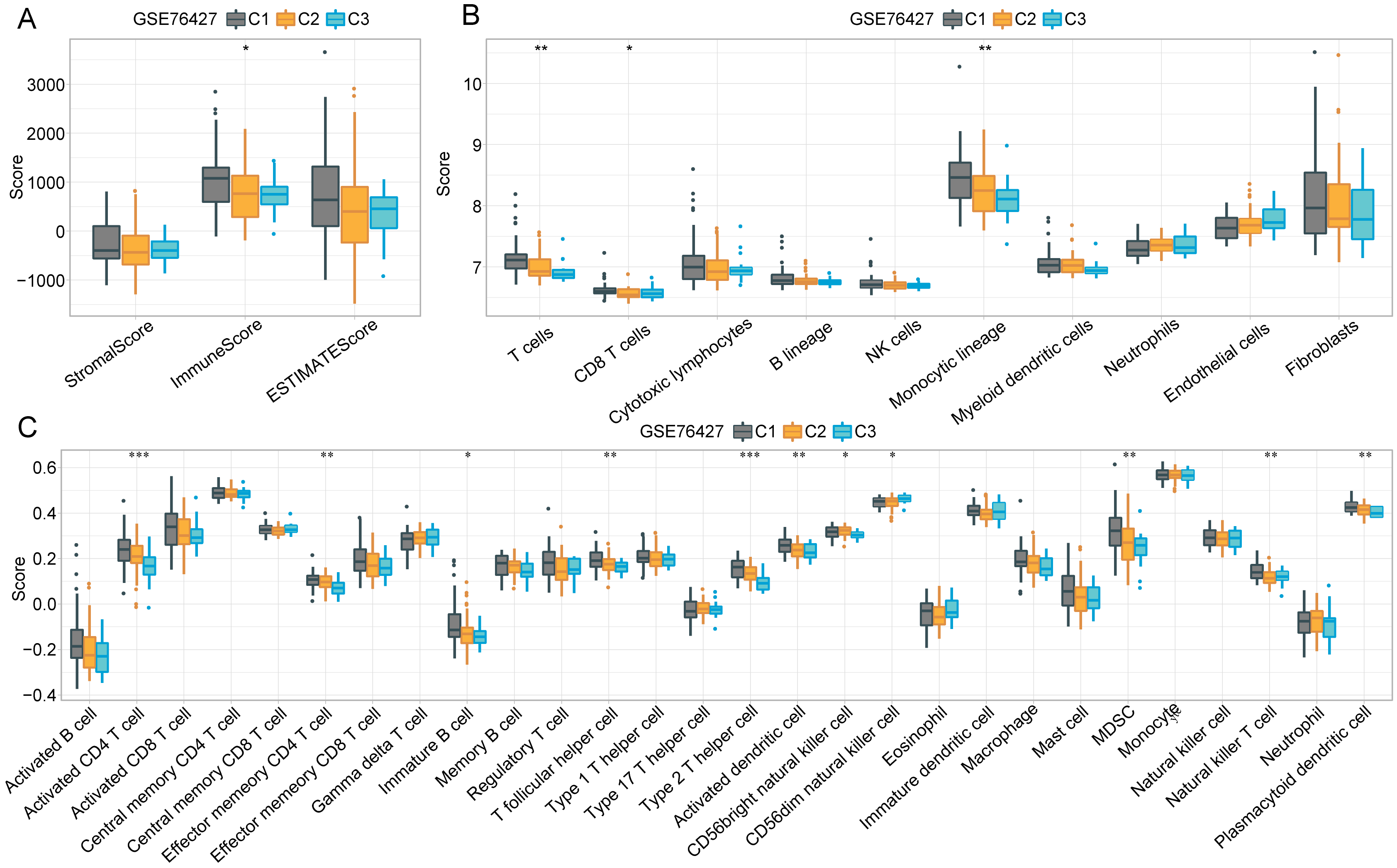

Supplement: Supplementary file 3 — Supplementary Figure 2. [file 41598_2022_23681_MOESM3_ESM.tif]

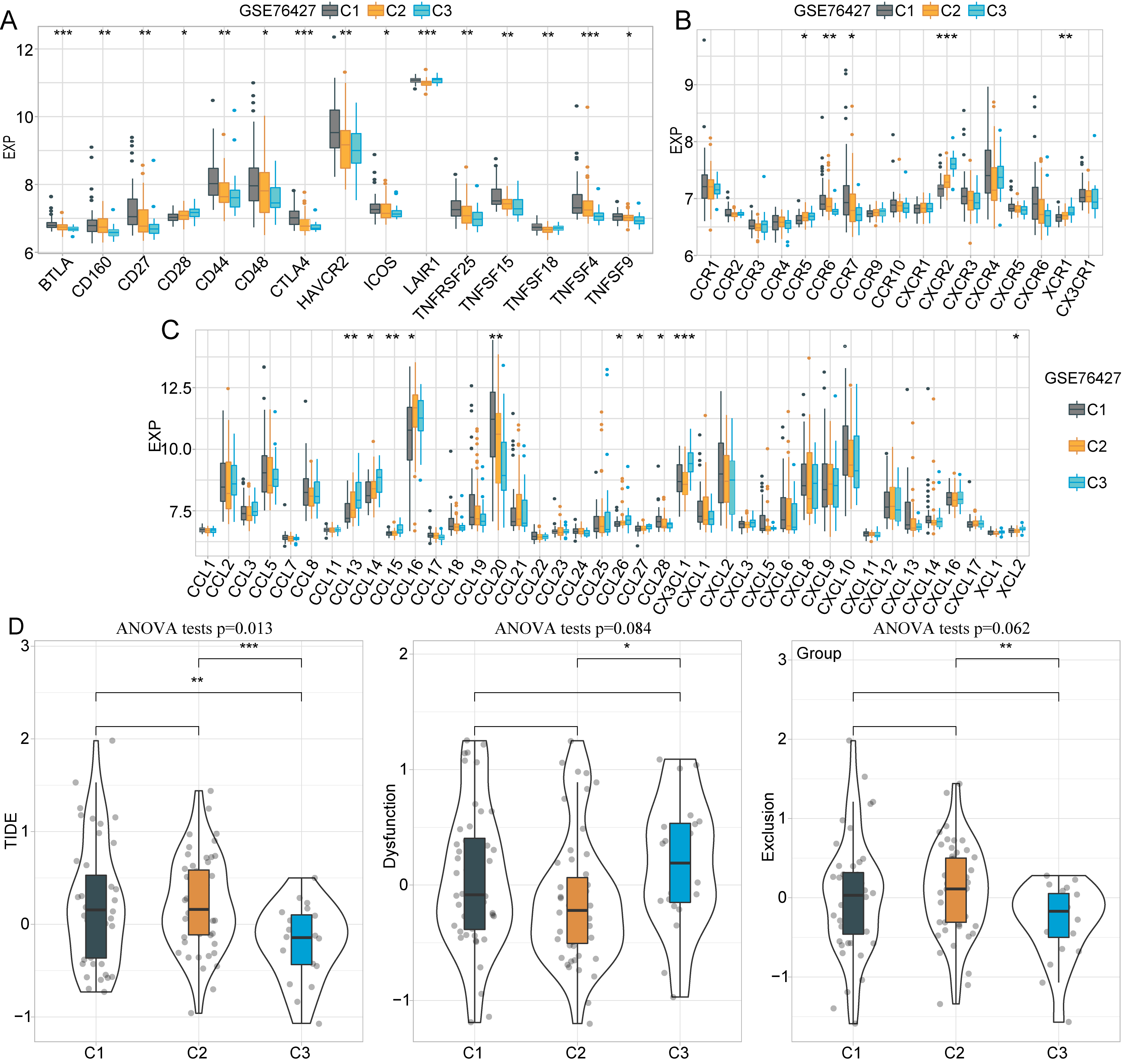

Supplement: Supplementary file 4 — Supplementary Figure 3. [file 41598_2022_23681_MOESM4_ESM.tif]

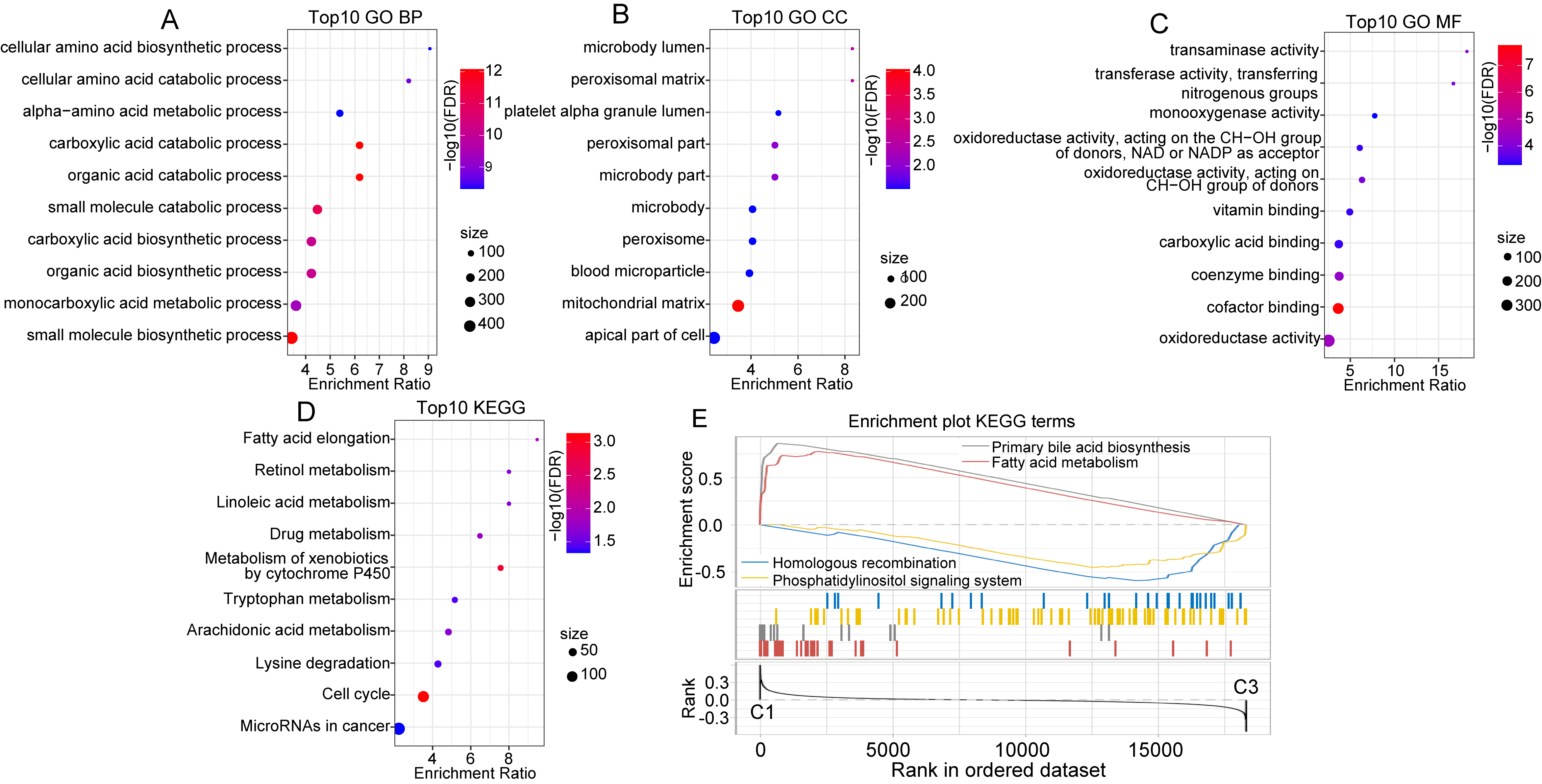

Supplement: Supplementary file 5 — Supplementary Figure 4. [file 41598_2022_23681_MOESM5_ESM.tif]

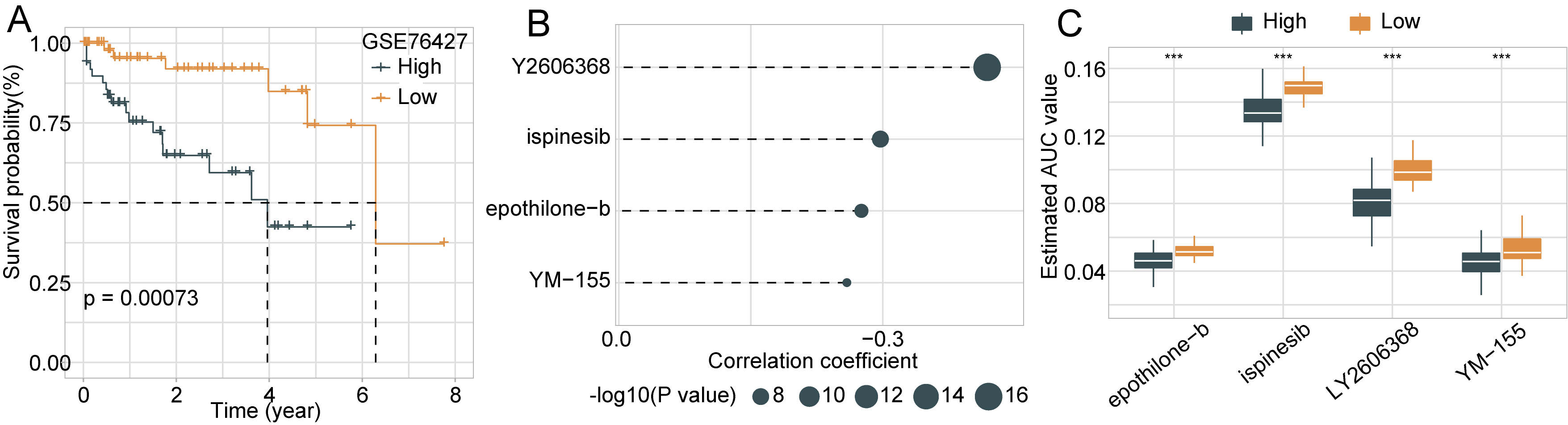

Supplement: Supplementary file 6 — Supplementary Figure 5. [file 41598_2022_23681_MOESM6_ESM.tif]
