# Supplementary material for: A novel classification of HCC basing on fatty-acid-associated lncRNA
Source: Sci Rep. 2022 Nov 7;12:18863. doi: 10.1038/s41598-022-23681-0 (PMC9640627; doi:10.1038/s41598-022-23681-0)
Supplement: Supplementary file 7 — Supplementary Tables. [file 41598_2022_23681_MOESM7_ESM.docx]

**Supplementary Table Legend**

**Supplementary Table 1** Seven key lncRNAs related to fatty-acid.

**Supplementary Table 2** The information of primers sequences of 9 lncRNAs

**Supplementary Table 1** Seven key lncRNAs related to fatty-acid.

| **ENSGID** | **Genes** | **beta** | **HR** | **lower 95%** | **upper 95%** | **P-value** |
| --- | --- | --- | --- | --- | --- | --- |
| ENSG00000231889 | TRAF3IP2-AS1 | 0.693688 | 2.0011 | 1.0702 | 3.742 | 0.0298 |
| ENSG00000247092 | SNHG10 | 0.015462 | 1.0156 | 0.7493 | 1.376 | 0.9206 |
| ENSG00000234091 | AL157392.2 | 0.242901 | 1.2749 | 0.9738 | 1.669 | 0.0772 |
| ENSG00000230131 | LINC02641 | 0.198987 | 1.2202 | 0.5319 | 2.799 | 0.6385 |
| ENSG00000237950 | AL357079.1 | 0.363394 | 1.4382 | 1.0207 | 2.027 | 0.0378 |
| ENSG00000248932 | AC046134.2 | -0.0018 | 0.9982 | 0.6448 | 1.545 | 0.9936 |
| ENSG00000268895 | A1BG-AS1 | -0.25215 | 0.7771 | 0.5675 | 1.064 | 0.1158 |

**Supplementary Table 2**

| **Primer name** | **Sequence** |
| --- | --- |
| AC012499.1 | F: CACACCATTCTCCTGCCTCA |
|  | R: GGATTAGGGAGGGAGGAGTTT |
| DRAIC | F: GTCACCTTGCGTGGAGTATG |
|  | R: TCTTAATGCCTTCAACTGATTGGA |
| LINC01951 | F: TGGGATTATAGGCGTGAACTA |
|  | R: CAATACTTCAGCACTAACCACC |
| LINC01625 | F: CCGATTGTTGGAGAAGTGGAA |
|  | R: CCGATTGTTGGAGTCAATT |
| AF127577.4 | F: GTAAGTGTGCTGATTCGTAGAG |
|  | R: TCCTACTGCGTGTCTAATGAAC |
| ACO68631.1 | F: GTGCCTGGAGTGAAGATTCTG |
|  | R: CCTAAGGTCCTGCGAGTACA |
| LINC01124 | F: ATGTTCATCTGTGCCATCTGTA |
|  | R: TCTGTGCCAAATGCTGTAGTAG |
| AP003498.1 | F: CTGTGTCTGCCATCTTCATCAT |
|  | R: CTGTAGTTCAAAGTGCCTATCC |
| AC005100.1 | F: GAGGGAATGGCACTTGGAAA |
|  | R: AAGCCACAGCAGACTTAGGT |
